# Cryopreservation and post-thaw characterization of dissociated human islet cells

**DOI:** 10.1371/journal.pone.0263005

**Published:** 2022-01-26

**Authors:** Leah A. Marquez-Curtis, Xiao-Qing Dai, Yan Hang, Jonathan Y. Lam, James Lyon, Jocelyn E. Manning Fox, Locksley E. McGann, Patrick E. MacDonald, Seung K. Kim, Janet A. W. Elliott

**Affiliations:** 1 Department of Chemical and Materials Engineering, University of Alberta, Edmonton, Alberta, Canada; 2 Department of Laboratory Medicine and Pathology, University of Alberta, Edmonton, Alberta, Canada; 3 Department of Pharmacology and the Alberta Diabetes Institute, University of Alberta, Edmonton, Alberta, Canada; 4 Department of Developmental Biology, Stanford University School of Medicine, Stanford, CA, United States of America; 5 Stanford Diabetes Research Center, Stanford University School of Medicine, Stanford, CA, United States of America; 6 Endocrinology Division, Department of Medicine, Stanford University School of Medicine, Stanford, CA, United States of America; University of Maryland School of Medicine, UNITED STATES

## Abstract

The objective of this study is to optimize the cryopreservation of dissociated islet cells and obtain functional cells that can be used in single-cell transcriptome studies on the pathology and treatment of diabetes. Using an iterative graded freezing approach we obtained viable cells after cooling in 10% dimethyl sulfoxide and 6% hydroxyethyl starch at 1°C/min to –40°C, storage in liquid nitrogen, rapid thaw, and removal of cryoprotectants by serial dilution. The expression of epithelial cell adhesion molecule declined immediately after thaw, but recovered after overnight incubation, while that of an endocrine cell marker (HPi2) remained high after cryopreservation. Patch-clamp electrophysiology revealed differences in channel activities and exocytosis of various islet cell types; however, exocytotic responses, and the biophysical properties of voltage-gated Na^+^ and Ca^2+^ channels, are sustained after cryopreservation. Single-cell RNA sequencing indicates that overall transcriptome and crucial exocytosis genes are comparable between fresh and cryopreserved dispersed human islet cells. Thus, we report an optimized procedure for cryopreserving dispersed islet cells that maintained their membrane integrity, along with their molecular and functional phenotypes. Our findings will not only provide a ready source of cells for investigating cellular mechanisms in diabetes but also for bio-engineering pseudo-islets and islet sheets for modeling studies and potential transplant applications.

## Introduction

Cryopreservation is an enabling technology providing on-demand access and distribution of biological material (cells and tissues) for collaborative, multimodal, and interdisciplinary studies. For example, investigations using single-cell genomics and electrophysiology to characterize cells in the human body will enhance our understanding of the cellular mechanisms of health and disease [1,2]. In this work, we focus on optimizing the cryopreservation of dispersed islet cells from human pancreas. The ability to store islet cells long-term and obtain cells whose transcriptomes and functions are retained upon thawing will deliver a useful and repeatedly accessible cell source for basic cellular and molecular research on the pathology and treatment of diabetes, and for potential transplant applications.

It was estimated that in 2019 there were 463 million people with diabetes worldwide and this number is expected to increase to 700 million by 2045 [3]. In the United States in 2018, approximately 34.2 million people of all ages had diabetes [4]. Elucidating the mechanisms underlying diabetes is hampered by limitations in the availability and accessibility of human islet cells for single-cell characterization studies such as patch clamp electrophysiology and single-cell RNA sequencing (scRNA-seq). Cryopreservation will allow the availability and distribution of dissociated islet cells for these investigations.

Native islets are multi-cellular structures, which makes them more challenging to cryopreserve than single cells because of non-uniform water and cryoprotectant content in the core cells compared to cells on the outer layer during freezing. Islets have a mean diameter of 150 μm each comprising 1,000–10,000 cells including α, β, δ, ε, and PP cell types that secrete glucagon, insulin, somatostatin, ghrelin, and pancreatic polypeptide, respectively [5,6]. An understanding of what occurs at the cellular level during freezing, and optimization of the cryopreservation of dispersed single cells may be leveraged to increase cryopreservation survival of whole islets. Pseudoislets that are custom-designed with varying numbers and proportions of the desired cell types [7,8] or containing genetically modified cells [9] have emerged as model systems for investigating signalling pathways and hormone secretion in diabetes [10–13]. Furthermore, it has been demonstrated in an animal model that transplantation of cryopreserved single islet cells that are re-aggregated into their natural spheroid form after thawing resulted in a better outcome compared to transplantation with fresh native islets [14]. Thus, an optimized procedure for the cryopreservation of dissociated islet cells may be beneficial in diabetes research and in improving transplantation outcomes.

There are critical sub-zero temperature ranges where interrupting the cooling process improves cell recovery. Here, we applied an interrupted slow cooling procedure called graded freezing to study the injury that dispersed islet cells incur when they go through the freezing process [15–18]. As cells in aqueous solution are cooled, ice forms in the surroundings thereby increasing the extracellular concentration of solutes. In response, water leaves the cells. If the cooling rate is too low, cells experience a severe volume shrinkage and prolonged exposure to high solute concentrations; this is referred to as slow-cooling injury. In contrast, if cells are cooled rapidly, the intracellular water does not have time to equilibrate with the extracellular solution and may cause ice to form inside the cells (intracellular ice formation); this is called rapid-cooling injury [19]. In graded freezing, the response of cells is studied as a function of temperature throughout a cooling protocol. At various temperatures along the slow-cooling profile (e.g. 1°C/min), cells are either directly thawed and assessed for membrane integrity to gain information about slow-cooling injury, or plunged into liquid nitrogen and then thawed and assessed in order to identify rapid-cooling injury. Further experiments can be designed to mitigate the freezing injury by employing optimal cooling rates or by the addition of cryoprotectants. Permeating cryoprotectants, such as dimethyl sulfoxide (DMSO), increase intracellular and extracellular osmolality, depress the freezing temperature, and reduce the amount of ice formed at a given temperature thereby minimizing cell shrinkage and the extent of slow-cooling injury [20]. Non-permeating cryoprotectants such as hydroxyethyl starch (HES), increase extracellular osmolality, drawing water out of cells earlier in the cooling profile, thus reducing the likelihood of intracellular ice formation and minimizing rapid cooling injury [21].

Optimization of the cryopreservation protocol was achieved using an iterative graded freezing approach that examined the effects of cooling profile parameters, cryoprotectant type and concentrations, cooling rate, and the presence of HEPES buffer. The procedure that yielded cells with the highest membrane integrity post-thaw was used to cryopreserve dispersed islet cells for patch clamp electrophysiology and scRNA-seq. Patch-clamp electrophysiology allows the measurement of changes in membrane potential as vesicles fuse with the cell membrane and secrete their contents during exocytosis, while scRNA-seq profiles the molecular identity of individual cells from their gene expression. By performing these two techniques (patch-seq) on the same cell it is possible to link endocrine physiology and transcriptomes at the single-cell level [22]. Here, the viability and cellular composition of dispersed islet cells before and after cryopreservation were compared. We also examined the exocytosis response (total exocytosis, rapid and sustained exocytosis), ion channel activities of individual cell types, and transcriptome profiles of fresh islet cells and thawed cells cryopreserved using the optimized protocol.

## Materials and methods

### Isolation of islets and dispersion into single cells

Human pancreatic islets were obtained from 27 deceased organ donors (18 male and 9 female; mean age 46.6 years, range: 14–74 years old) after informed consent for use of pancreatic tissue for research (Table 1). Written informed consent for research use of pancreas was obtained from donors’ next of kin, and organ donation was coordinated by the Human Organ Procurement and Exchange program in Edmonton, with the Trillium Gift of Life Network (www.giftoflife.on.ca) and other Canadian organ procurement organizations (ref: https://doi.org/10.1210/en.2015-1562, www.bcell.org/adi-isletcore). Obtaining and processing organs for islet studies was approved by the Human Research Ethics Board at the University of Alberta (Pro00001754, Pro00013094). The islets were isolated by the Alberta Diabetes Institute IsletCore at the University of Alberta as previously described [23,24]. The purity of the preparation was assessed by dithizone staining of endocrine tissue and the average percent purity of the islet samples used in this study was 80% (range: 50–90%).

**Table 1:** Characteristics of pancreatic islet donors.

Record ID#	Donor Age	Sex (M = 18; F = 9)	BMI	HbA1c	Purity %	Culture time (h)	Donor islets used for
R266	74	F	29.2	6.0	90	80	Fig 1A
R267	64	F	23.7	6.3	90	87	Fig 1A
R268	48	F	29.2	5.4	90	20	Fig 1A
R269	14	M	21.5	NA	50	45	Fig 1A
R270	35	F	25.5	5.3	75	68	Fig 1B
R271	60	F	26	5.5	95	90	Fig 1B
R272	56	M	26.9	5.6	75	14, 38, 62	Fig 1A and 1B
R273	53	M	31.6	NA	75	42	Fig 1B
R274	42	M	25.2	NA	75	44, 68, 92	Figs 2A and S3
R275	46	M	28.7	4.5	75	18, 42	Figs 1A and S3
R276	54	F	24.4	7.2	75	20, 44, 68	Figs 1B and 2A (2x)
R277	47	M	33.8	5.6	75	18	S3 Fig
R280	17	F	26.5	5.3	50	68	Fig 1A
R282	57	M	26.4	6.0	90	39	Fig 2B
R283	22	M	22.5	NA	90	15, 39	Fig 2B
R292	47	M	27.6	5.6	90	17	Figs 3A, 3B, 4 and 5
R297	69	M	27.2	NA	95	12	Figs 3A, 3B, 4 and 5
R305	60	M	21.4	5.6	80	84	Figs 3A, 3B, 4 and 5
R310	25	M	26.4	5.4	90	89	Fig 1B
R314	31	F	30.3	5	80	12	Fig 1B
R316	52	M	26	5.7	50	88	Fig 1B
R317	54	M	26.4	5.1	90	64	Fig 1B
R318	54	M	20.5	5	90	16	Fig 1B
R322	44	F	23.2	4.9	90	17	Fig 2B
R324	58	M	36.6	5.1	75	16	Fig 2B
R325	50	M	30.3	NA	75	18	Fig 2B
R326	26	M	27	5.5	90	35	Fig 2B
Mean	46.6		26.8	5.5	80%	42 h	
Range	14 to 74		20.5 to 36.6	4.5 to 7.2	50 to 95%	12 to 92 h	

Islets were cultured in CMRL 1066 medium (Corning Inc., Oneonta, NY) supplemented with 0.5% bovine serum albumin (Equitech-Bio Inc., Kerrville, TX), 1% insulin-transferrin-selenium (Corning), 100 U/mL penicillin/streptomycin (Life Technologies Corp., Carlsbad, CA), and L-glutamine (Sigma-Aldrich, Oakville, ON) (complete CMRL medium) in a humidified incubator at 22°C and 5% CO_2_ (culture period range: 12–90 h). Handpicked islets were dispersed into single cells by first centrifuging at 120 x g for 1 min. The medium was aspirated, and then 1 mL TrypLE Express (Gibco, ThermoFisher Scientific, Waltham, MA) was added and the pellet re-suspended. Our choice of TrypLE as the islet dissociation reagent was based on an earlier study [25] which compared various enzymatic cell dissociation agents namely Trypsin, TrypLE, Accutase, Dispase, Papain and Accumax (a collagenase-containing reagent) to dissociate native islets, where TrypLE showed the highest percentage of recovered viable cells. DNase solution (20 μg/mL, StemCell Technologies, Vancouver, BC) was added to minimize cell clumping. The islets were placed in a 37°C water bath for 10 min, followed by gentle pipetting up and down until islets were visibly dispersed and no clumps remained. DMEM (Gibco, 11885–084) supplemented with 10% fetal bovine serum and 100 U/mL penicillin/streptomycin (complete DMEM) was added (9 mL) and the cells were centrifuged for 3 min at 200 x g. The supernatant was removed and the pellet of dispersed islet cells was re-suspended in an appropriate volume of complete DMEM, assessed for membrane integrity, and used for downstream experiments.

### Graded freezing

Graded freezing was carried out as previously described [16–18]. Different cryoprotectant types (permeating: DMSO; and non-permeating: HES) and concentrations, cooling rates, and pH effects were examined. The cryoprotectants were prepared by weight in CMRL 1066 complete medium at double the final concentrations, and equal weights of the cell suspension and cryoprotectants were combined. HES (Preservation Solutions Inc., Elkhorn, WI) was prepared from a stock 20% Pentastarch Solution (200 mg/mL) in medium. The cryoprotectants were loaded by keeping the cell–cryoprotectant mixture on ice for 15 min. Aliquots of the cell suspension (0.2 mL) were transferred to 6 x 50 mm borosilicate glass test tubes (VWR, Edmonton, AB, Canada) and allowed to equilibrate for 2 min in a stirred methanol bath (FTS Systems Inc., Stone Ridge, NY, USA) pre-set at –5°C. Ice nucleation was induced using metal forceps cooled in liquid nitrogen, and the samples kept at –5°C for 3 min to allow the release of the latent heat of fusion. The methanol bath was then cooled at specific cooling rates to intermediate sub-zero temperatures (–10°C, –20°C, –30°C, –40°C, and –50°C), as the temperature was monitored by a T-type thermocouple and OMB-DAQ-55 data acquisition module and Personal Daq View software (OMEGA Engineering Inc., Stamford, CT). At each experimental temperature, one set of duplicate samples (total volume: 400 μL) was thawed directly in a 37°C water bath (direct-thaw samples) and another set was plunged into liquid nitrogen (plunge-thaw samples). The plunged samples were kept in liquid nitrogen for at least 1 h before rapid thawing in a 37°C water bath. After thawing, cells were immediately stained for membrane integrity assessment by flow cytometric analysis.

### Membrane integrity assessment

A dual fluorescent stain (SYTO 13/GelRed) containing SYTO 13 (Molecular Probes, Eugene, OR) and GelRed (Biotium, Scarborough, ON) was prepared fresh from stock solutions of (15 μL of 5 mM SYTO 13 and 25 μL of 10000x GelRed in 279 μL water) on the day of the experiment. Twenty µL of stain was added to each 400 μL sample, and then the samples were incubated for 10 min at room temperature in darkness prior to being run on an Epics XL-MCL flow cytometer (Beckman Coulter Inc., Pasadena, CA). The fluorescence color compensation was adjusted to 32% and optimized flow cytometer settings were: forward scatter voltage 150, gain 2; side scatter voltage 550, gain 10; FL1 voltage 300, gain 7.5; FL3 voltage 315, gain 7.5 [26]. SYTO 13 penetrates the cell membranes of all cells and complexes with nucleic acids to emit a green fluorescence when excited by ultraviolet light [27]. GelRed penetrates only cells with damaged membranes and emits an intense red fluorescence. When cells stain positive for GelRed the SYTO 13 signal is quenched through a variety of mechanisms [27,28]. Therefore, SYTO 13-labeled cells (green) are considered to have intact membranes and consequently to be viable, while GelRed-labeled (red) and GelRed/SYTO 13 double-stained cells are considered to have compromised membranes and counted as non-viable. A representative dot plot is shown in S1 Fig. Flow cytometry results were analyzed using the Kaluza Analysis software (version 1.3) from Beckman-Coulter. The percent membrane integrity was calculated from the ratio of the number of membrane-intact cells (counted from the SYTO 13 quadrant) to the total number of cells (counted from the SYTO 13, GelRed, and double-stained quadrants).

### Effect of cryoprotectant removal and extended incubation on membrane integrity

Dispersed islet cells were cooled at 1°C/min in the presence of 10% (w/w) DMSO and 6% (w/w) HES to –40°C before plunging into liquid nitrogen. The cells were thawed in a 37°C water bath. Membrane integrity was assessed as described above under the following conditions: i) no cryoprotectant removal (cells were immediately stained with SYTO 13/GelRed); ii) immediate cryoprotectant removal (cells were centrifuged for 200 x g for 5 min, supernatant was removed, and the cells re-suspended in 400 μL PBS before staining); iii) one-step dilution (4x the volume of cell suspension of PBS was added, cells were centrifuged for 200 x g for 5 min, supernatant was removed and the cells re-suspended in 400 μL PBS before staining); and iv) serial dilution (400 μL PBS plus 20% fetal bovine serum (FBS, Gibco) was added and cells were incubated for 2 min at room temperature, then 400 μL PBS plus 10% FBS was added and cells were incubated for 2 min at room temperature, this step was repeated 3 times, then cells were centrifuged for 200 x g for 5 min, supernatant was removed and the cells re-suspended in 400 μL PBS before staining).

For removal of cryoprotectants from dispersed islet cells in cryovials post-thaw, the contents of the cryovial (~ 1 mL) were transferred to a 15-mL centrifuge tube and serial dilution was performed as described above with volumes adjusted proportionately. After the final centrifugation, the supernatant was removed and the cells were re-suspended in complete DMEM and used for fluorescence-activated cell sorting (FACS), electrophysiological phenotyping, and scRNA-seq, as described below. In some experiments, the membrane integrity of dispersed islet cells was assessed after overnight incubation in complete CMRL medium at 37°C and 5% CO_2_.

### FACS analysis

Table 1 shows the clinical characteristics of the three pancreatic donors (R292, R297, and R305) whose islets were used to compare fresh vs. cryopreserved islet cells. Dispersed single cells from fresh or cryopreserved human islets were first stained with LIVE/DEAD Fixable near-IR dead cell dye (Life Technologies, L10119) as a viability marker. This is an amine reactive dye that binds covalently to intracellular and extracellular amines in cells with compromised membranes, and whose reactivity is restricted to amines on the cell surface in viable cells [27]. Thus, membrane-damaged cells give rise to intense fluorescent staining; in contrast, viable cells yield relatively dim staining. Prior to antibody staining, cells were blocked with mouse IgG in FACS buffer (2% FBS, 10mM EGTA, in PBS). The following antibodies were used for FACS experiments at 1:100 (v/v) final concentration, CD31-FITC (BioLegend, 303103, San Diego, CA), CD45-PE/Dazzle594 (BioLegend, 304052), CD90-Alexa Fluor 700 (BioLegend, 328120), EpCAM-APC (BioLegend, 324208), HPi2-Alexa405 (Novus Biologicals, NBP1-18946AF405, Centennial, CO), and HPx1-PE (Novus Biologicals, NBP1-18951PE). All antibody incubation steps were performed on ice for 30 minutes. Labeled cells were sorted on a special order 5-laser FACS Aria II (BD Biosciences, San Jose, CA) with cytometry data analyzed and graphed using FlowJo software (TreeStar v.10.8).

### Electrophysiological phenotyping

Fresh dispersed islet cells and their cryopreserved counterparts (from donors R292, R297, and R305) were cultured in low glucose (5.5 mM) DMEM with L-glutamine, 110 mg/L sodium pyruvate, 10% FBS, and 100 U/mL penicillin/streptomycin for 1–3 days. The medium was then changed to bath solution containing: 118 mM NaCl, 20 mM TEA, 5.6 mM KCl, 1.2 mM MgCl_2_, 2.6 mM CaCl_2_, 5 mM HEPES, and 5 mM glucose (pH adjusted to 7.4 with NaOH) in a heated chamber (32–35°C). For whole-cell patch-clamping, fire polished thin wall borosilicate pipettes coated with Sylgard (3–5 MOhm) contained intracellular solution (125 mM Cs-glutamate, 10 mM CsCl, 10 mM NaCl, 1 mM MgCl_2_, 0.05 mM EGTA, 5 mM HEPES, 0.1 mM cAMP and 3 mM MgATP (pH adjusted to 7.15 with CsOH)). Single cell exocytosis was measured as described previously [29]. Ca^2+^ charge entry was obtained by a single depolarization from –70 to 0 mV. Voltage-gated Na^+^ and Ca^2+^ channel currents were measured by membrane depolarization from –60 to +30 mV. Steady-state inactivation of Na^+^ channel was measured by a two-pulse protocol, in which cells were held at pre-pulse potentials ranging from –120 to –10 mV for 200 ms and then subjected to a –10 mV test pulse for 25 ms. Reversal potential was measured by a voltage ramp protocol from –120 mV to +60 mV, while the hyperpolarization-activated non-selective cation currents were measured by membrane hyperpolarization of –140 mV. Electrophysiological measurements were collected using a HEKA EPC10 amplifier and PatchMaster Software (HEKA Instruments Inc., Lambrecht/Pfalz, Germany) within 5 minutes of break-in. Quality control was assessed by the stability of seal (>10 GOhm) and access resistance. These protocols are schematically shown in S2 Fig. The number of fresh cells (32, 24, and 40) and cryopreserved cells (32, 40, and 40) used for electrophysiology are from donors R292, R297, and R305, respectively. Within minutes after patching cells, we directly collected the whole cellular content with a secondary pipette filled with lysis buffer for the SmartSeq2 RNASeq procedure [22]. Of the total 208 cells that were patch-sequenced, 189 (91%) passed quality control for both electrophysiology and sequencing.

### Single cell RNA-sequencing

We generated cDNA and sequencing libraries using the SmartSeq-2 protocol [30]. Patch-clamped single cells were assembled into 96-well plates containing lysis buffer as previously described [31]. Briefly, mRNAs were primed with an anchored oligo-dT and reverse transcribed using an LNA-containing template switching oligo, followed by PCR amplification (23 cycles). Libraries were then generated from the amplified cDNA by tagmentation with Tn5. Libraries were sequenced in a NovaSeq platform (Illumina, San Diego, CA) using paired-end reads (100 bp) to an average depth of 1 million reads per cell. Sequencing reads were aligned to the human genome (GRCh38 genome with supplementary ERCC sequences) using STAR [32], and gene counts determined using htseq-count (intersection-nonempty) using a GTF annotation with Ensembl 97 release genes [33]. Gene expression was normalized to counts per million (cpm) after removal of counts corresponding to ERCC spike-ins and transformed to natural log values after addition of a pseudocount. Data analysis was performed using Seurat v3.1.1 [34] unless otherwise noted.

### Statistical analysis

For the graded freezing experiments, statistical significance was defined at 95% confidence level and calculated using paired, two-tailed, Student’s t-tests. Statistical calculations were carried out using Microsoft Excel 2016 (Microsoft Corporation, Redmond, Washington, United States). Electrophysiological data were expressed as mean and standard error. When comparing two groups we used Student’s t-tests. When comparing more than two groups we used one-way or two-way ANOVA followed by the Tukey post-test. P-values less than 0.05 were considered to be significant. Differential expression analysis of single cell RNA sequencing data was performed using the “model-based analysis of single-cell transcriptomics” method as previously described [35]. The p-values were adjusted based on Bonferroni correction using all features remaining after QC filtering.

## Results

### Optimization of cryopreservation of dispersed islet cells

**Fig 1:**
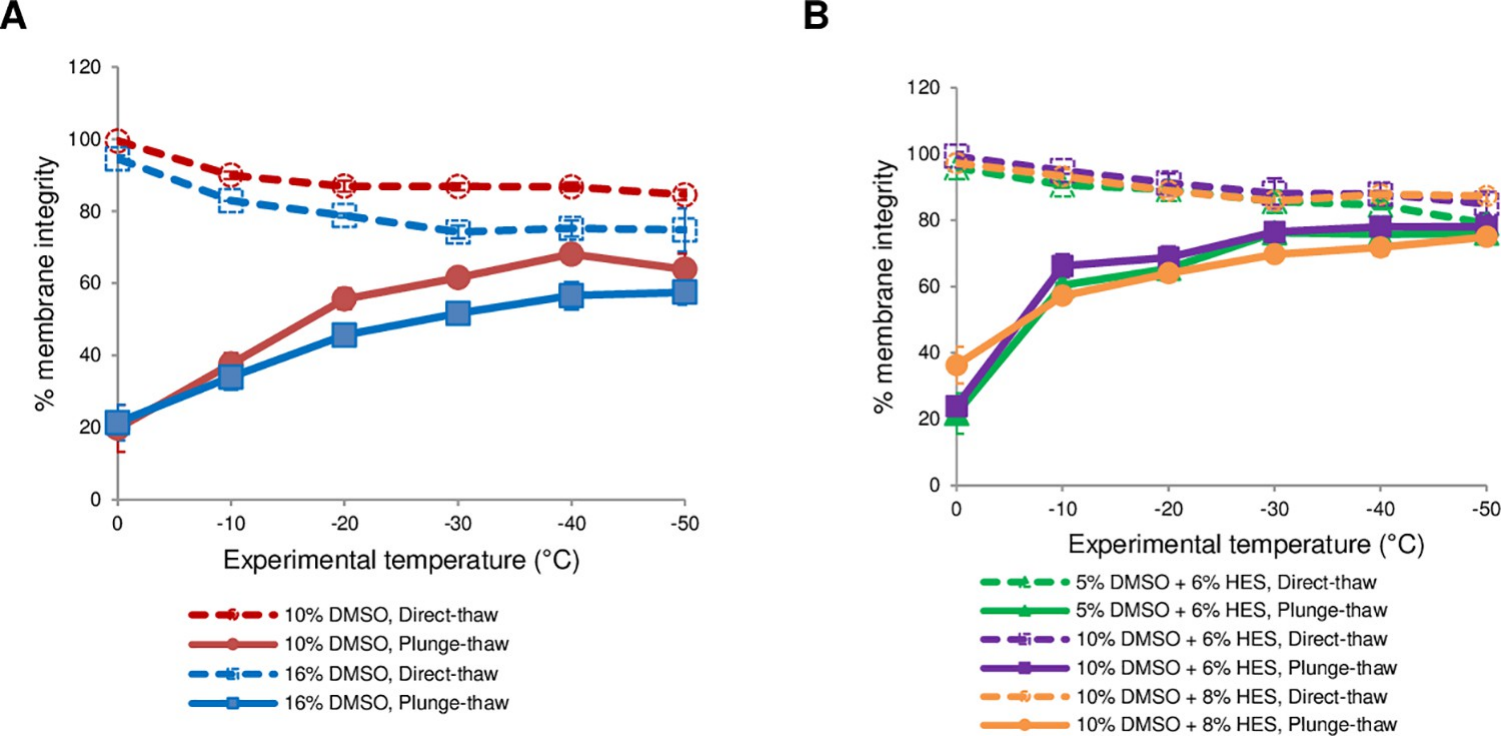
Membrane integrity of dispersed islet cells subjected to graded freezing. The cells were cooled to various sub-zero temperatures in the presence of different concentrations of cryoprotectants (DMSO: Dimethyl sulfoxide; HES: Hydroxyethyl starch) at 1°C/min. The samples were either directly thawed (open symbols, broken lines) or plunged and stored in liquid nitrogen before rapid thawing (solid symbols and lines). The percent membrane integrity was normalized against live unfrozen control. Data points are the mean of independent experiments (N values) and error bars represent standard error of the mean. (**A**) Comparison between 10% DMSO (N = 3) and 16% DMSO (N = 2). (**B**) Comparison between 5% DMSO + 6% HES (N = 2), 10% DMSO + 6% HES (N = 3), and 10% DMSO + 8% HES (N = 2) (S1 Table).

The graded freezing technique enables optimization of cryopreservation procedures by delineating and mitigating cell injury that occurs upon slow cooling to intermediate sub-zero temperatures (direct-thaw samples) from that which happens during rapid cooling to the liquid nitrogen storage temperature (plunge-thaw samples). The cell damage during cooling was assessed by flow cytometric analysis of membrane integrity because the plasma membrane is a major site of cryoinjury [36]. The cell response along the cooling profile is depicted in Fig 1. In both panels (A and B), the broken lines represent the viability of dispersed islet cells that were directly thawed and the solid lines represent the viability of samples that were plunge-thawed. Under all conditions, the percent membrane integrity was over 95% at the beginning of the cooling protocol, after cells were allowed to equilibrate with the cryoprotectants at 0°C for 15 min. DMSO at 10% (w/w) concentration was able to mitigate slow cooling injury in dispersed islet cells as demonstrated by the high viability of direct-thaw samples (Fig 1, panel A, broken red line). However, it is worth noting that the membrane integrity of direct-thaw samples declined steadily from 99.4 ± 0.3 at 0°C to 84.5 ±1.4 at –50°C in the presence of 10% DMSO. It is possible that DMSO at 10% is not sufficient to mitigate slow-cooling injury. Yet, increasing its concentration to 16% did not improve the membrane integrity of direct-thaw samples which instead went down to 74.8 ± 6.0 at –50°C (Fig 1, panel A, broken blue line). Cryoprotectants can cause osmotic shifts that can be damaging to cell membranes, and depending on concentration, can be toxic. Thus, 16% DMSO did not provide any additional benefit towards mitigating slow-cooling injury in cryopreserved dispersed islet cells, and may have instead contributed to cellular toxicity. Still, 10% DMSO conferred some protection against rapid cooling injury by permeating the cells and likely minimizing the amount of intracellular ice formed as previously shown [37,38], resulting in 68.1 ± 1.9% membrane integrity after plunging into liquid nitrogen from –40°C and then thawing (Fig 1, panel A, solid red line). We found that increasing the concentration of DMSO to 16% did not further lessen rapid cooling injury along the entire cooling profile (Fig 1, panel A, solid blue line).

Based on our previous work on human umbilical vein endothelial cells in suspension, the procedure that yielded the highest post-thaw membrane integrity involves cooling cells at 1°C/min in the presence of 5% DMSO plus 6% HES [17]. The combination of 5% DMSO and 6% HES has been used in the cryopreservation of whole canine and human islets and has been shown to recover over 70% viable and fully functional islets [39]. Thus, in the next set of experiments the DMSO concentration was reduced to 5%, and 6% of the non-permeating cryoprotectant HES was added (Fig 1, panel B, green lines). HES was able to protect against intracellular ice formation and this was manifested in the higher membrane integrities along the cooling profile in comparison to using 10% DMSO alone. The membrane integrity of the plunge-thaw samples increased from 21.7 ± 6.1% for plunge from 0°C to 76.0 ± 1.4% for plunge from –50°C (Fig 1, panel B, solid green line). However, it is evident from the direct-thaw samples that 5% DMSO was not sufficient to protect from solute effects injury as the membrane integrity of the direct-thaw samples steadily declined with decreasing temperature reaching 79.0 ± 2.6% at –50°C (Fig 1, panel B, broken green line). Therefore, we increased the DMSO concentration to 10% and retained the HES concentration at 6% (Fig 1, panel B, purple line). By doing so, we observed that solute effects injury was largely mitigated and 87.9 ± 3.2% membrane integrity was attained when samples were slowly cooled at 1°C/min and directly thawed from –40°C (Fig 1, panel B, broken purple line). Samples that were plunged into liquid nitrogen then thawed were increasingly protected from rapid cooling injury and reached the highest membrane integrity after plunge from –40°C in the presence of 10% DMSO and 6% HES of 78.1 ± 2.5% (Fig 1, panel B, solid purple line). This value is statistically different from the membrane integrity using 10% DMSO alone (p = 0.03), but not from the membrane integrity when using 5% DMSO + 6% HES for which the maximum viability after plunge-thaw was 76.0 ± 1.4% (p = 0.48). Increasing the concentration of HES to 8% in the presence of 10% DMSO resulted in lower membrane integrities for plunge-thaw samples (Fig 1, panel B, solid orange line). This response may be due to excessive dehydration wherein the cells shrink beyond a minimum tolerable cell volume.

We also investigated how reducing the cooling rate to 0.3°C/min affects membrane integrity. This resulted in lower membrane integrities particularly at the lower sub-zero experimental temperatures, which could be due to the longer exposure of cells to high solute concentrations (S3 Fig). It has been demonstrated that cooling rates, ranging from 0.25 to 75°C/min, did not have a major influence on the survival of whole islets when they were cooled in the presence of 2 M DMSO [40]. Because it has been recognized that pH decreases during hypothermic storage of mammalian cells and that the increase in concentration of hydrogen ions contributes to harmful cellular processes [41], we added HEPES buffer to our cryopreservation solution consisting of 10% DMSO plus 6% HES. HEPES has been shown to offer the best buffering efficiency due to its high buffer capacity at physiological pH [41]. Addition of 0.1 M HEPES to 10% DMSO and 6% HES improved the membrane integrity early on in the cooling profile, but did not confer additional protection at the lower sub-zero experimental temperatures (S3 Fig). Altogether, our iterative optimization approach indicated that cooling in 10% DMSO and 6% HES at a rate of 1°C/min to –40°C, then plunging and storing in liquid nitrogen is the best cryopreservation method for dispersed islet cells. Hereinafter, we used this procedure for all downstream assessments.

### Effect of cryoprotectant removal and extended incubation on membrane integrity assessed by SYTO 13/GelRed staining

**Fig 2:**
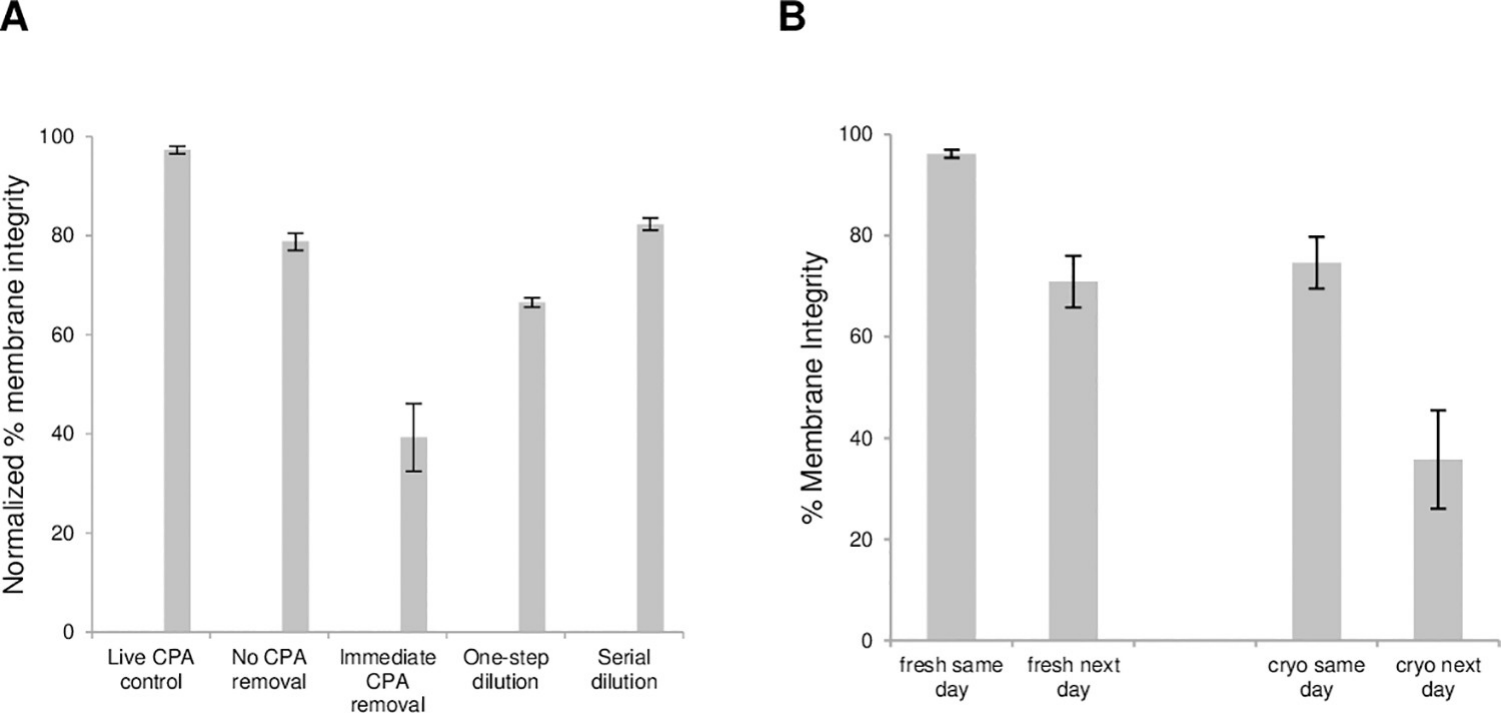
Effects of cryoprotectant removal (A) and extended incubation (B) on membrane integrity of dispersed islet cells. Islets were dispersed as described in the Materials and Methods and cooled at 1°C/min to –40°C in the presence of 10% DMSO and 6% HES, then stored in liquid nitrogen. Cells were thawed rapidly in a 37°C water bath. (A) Membrane integrity was assessed with a) no cryoprotectant (CPA) removal, b) immediate CPA removal, c) one-step dilution, and d) serial dilution, as described in the Materials and Methods. Fresh cells were used as positive control, and membrane integrity values were normalized against fresh cells in the absence of cryoprotectants. (B) For each donor sample, fresh and cryopreserved cells were assessed for membrane integrity on the same day or after overnight incubation in medium at 37°C and 5% CO_2_. Data points for both (A) and (B) are the average of three independent experiments and error bars represent standard error of the mean (S1 Table).

In the graded freezing experiments described above the membrane integrity was assessed immediately after thaw without removal of the cryoprotectants. In a separate set of experiments we found that when the best cryopreservation procedure was employed, the post-thaw cell viability remained high (78.8 ± 1.7%) if the cryoprotectant was not removed prior to membrane integrity assessment (Fig 2A). However, post-thaw cell cultures and functional assays often require cells in the absence of cryoprotectants. Therefore, membrane integrity was assessed after employing different methods of cryoprotectant removal (Fig 2A). When the cryoprotectants were immediately removed by centrifugation, the membrane integrity decreased (to 39.3 ± 6.9%). This was to be expected because of damaging osmotic transients that cells undergo due to unrestricted swelling as water moves into the cell faster than the DMSO can move out. By using a one-step dilution, the membrane integrity increased (to 66.5 ± 1.0%), and this was improved further (to 82.3 ± 1.2%) with the more gentle procedure of serial dilution. It is also an additional benefit that HES is present in the cryoprotectant solution, not only to stabilize the cell membrane, but also to reduce osmotic shock during DMSO removal [21]. Thus, for all cryopreserved cells in the following experiments the cryoprotectant was removed by serial dilution.

After removing the cryoprotectants, the dispersed islet cells were incubated overnight at 37°C. The membrane integrity of both fresh and cryopreserved cells was found to decrease significantly (from 96.2 ± 0.8% to 70.9 ± 5.1%, p = 0.008, and from 74.7 ± 5.1% to 35.8 ± 9.7%, p = 0.024, respectively) after overnight culture (Fig 2B). This is consistent with observations that some cellular damage by necrosis and apoptosis may not be evident immediately after thaw and could manifest itself many hours later even with proper cryoprotectant removal [42]. It has been claimed that cells may be membrane-intact when examined immediately after thawing; however, a significant portion (30–70%) of these cells may be membrane-damaged when examined 24–48 h later [42]. In fact, it has been reported for cryopreserved human islets that the percent of islet survival and quality post-thaw decreased with increasing number of days of culture, up to 7 days [43]. In addition to the cell viability (the ratio of membrane-intact cells to the total number of cells post-thaw), it is also important to take into account the cell recovery (the ratio of total live cells post-thaw to total live cells pre-freeze) after cryopreservation [44]. For the dispersed islet cells from the three samples (R292, R297 and R305) that were subjected to the best cryopreservation protocol, CPA removal by serial dilution, and used for electrophysiology and patch-seq gene profiling, the recovery was 84.7 ± 12.1%.

### Effect of cryopreservation and extended incubation on cell viability, cellular composition and surface marker expression

**Fig 3:**
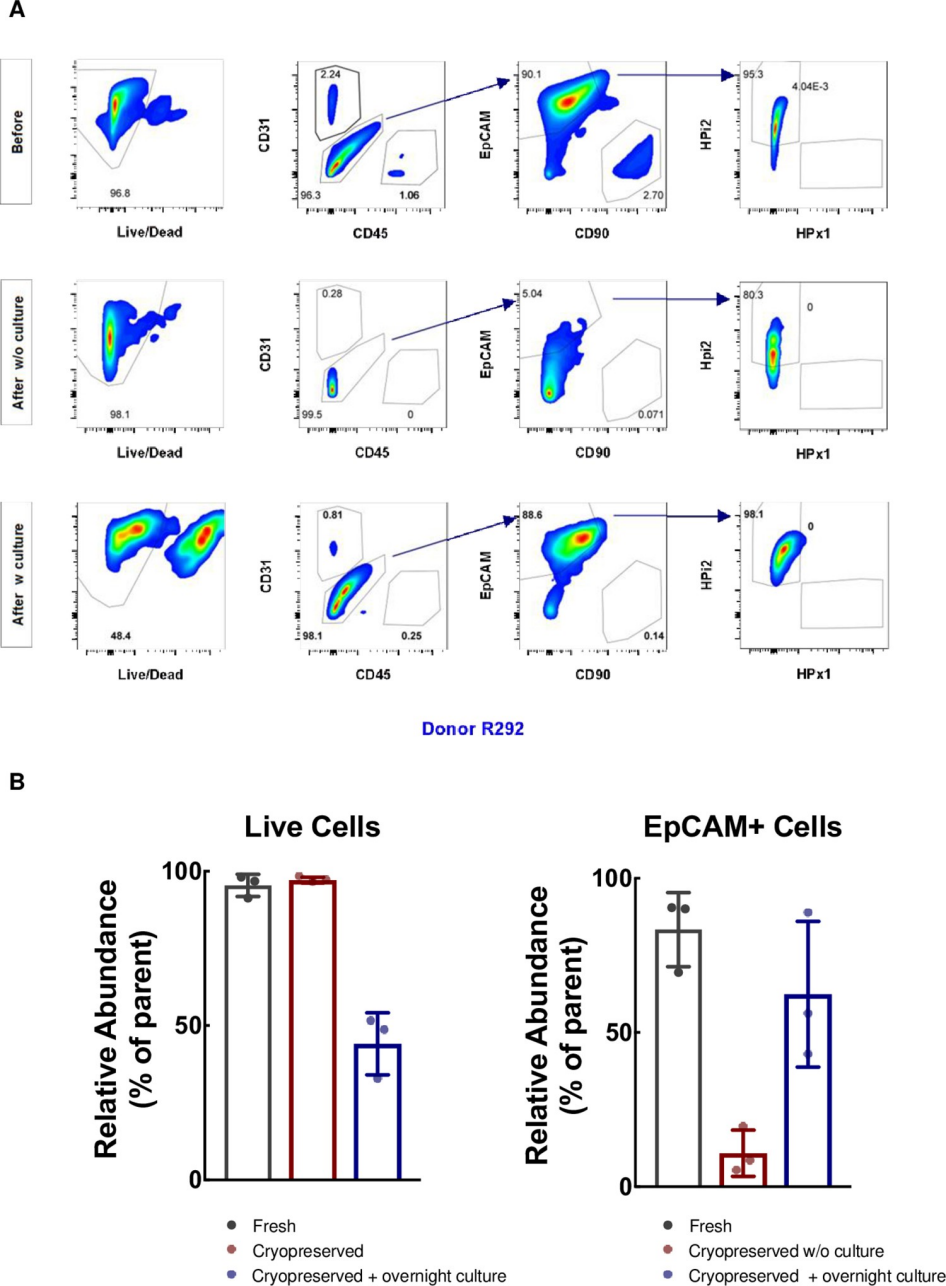
**(A) Representative FACS plots showing cell viability and the distribution of distinct pancreatic single islet cell populations.** Islets (from donor ID# R292) were dispersed as described in the Materials and Methods, and analyzed **before** cryopreservation (fresh, unfrozen control). Dispersed islet cells from the same donor were cooled at 1°C/min to –40°C in the presence of 10% DMSO and 6% HES, then stored in liquid nitrogen. Cells were thawed rapidly in a 37°C water bath, and the cryoprotectants were removed by serial dilution as described in the Materials and Methods. The cells were analyzed immediately (**after w/o culture)** or after overnight culture (**after w culture).** The cells were first stained with LIVE/DEAD Fixable near-IR dead cell dye to assess cell viability and analyzed using specific cell surface markers to stain distinct cell populations: CD31 for endothelial cells; CD45 for leukocytes; CD90 for mesenchymal cells; EpCAM for epithelial cells including acinar, ductal and islet cells; HPx1 for acinar cells and HPi2 for islet cells. FACS analyses were able to detect most of cell populations before after cryopreservation. **(B) Summary of FACS analysis of viability and EpCAM (surface marker for islet cells) expression in three pancreatic islet donors.** Fresh (black bars) and cryopreserved (red bars) dispersed islet cells from donors (see Table 1) were analyzed by flow cytometry. Cryopreserved cells were also cultured overnight and analyzed the next day (blue bars). Left panel: Cell viability is expressed as mean relative abundance ± standard error of the mean with actual data points represented as individual dots. Right panel: EpCAM expression.

To understand how the best method of cryopreservation and CPA removal affects islet cells, we used well-established markers in combination with flow cytometry to assess dispersed islets cells with or without cryopreservation. Islets from three donors (Table 1) were received unfrozen (and dispersed upon receipt at Stanford) or as optimally cryopreserved dispersed islet cells (i.e., cooled at 1°C/min to –40°C in the presence of 10% DMSO and 6% HES, stored in liquid nitrogen, then shipped in dry ice). Frozen cells were transferred and stored in liquid nitrogen until use, whereby cells were thawed rapidly in a 37°C water bath, and the cryoprotectants were removed by serial dilution. The cells were analyzed immediately or after overnight culture, and compared with their unfrozen counterparts. High viability is seen in both the fresh dispersed islet cells (95.4 ± 2.1%) and the cryopreserved post-thawed cells (97.1 ± 0.5%) (Fig 3A left-most histograms, Fig 3B left panel). As expected from high-purity islet preparations, there was a very low percent of CD31^+^ endothelial cells, CD45^+^ leukocytes, and CD90^+^ mesenchymal cells, and cryopreservation did not affect their distribution (Fig 3A). Epithelial cell adhesion molecule (EpCAM), a surface marker of epithelial cells including acinar, ductal, and islet cells, is detected in the majority of fresh cells (>80%). Among EpCAM^+^ epithelial cells, HPx1, an exocrine cell marker (of acinar cells) was at very low abundance in fresh and cryopreserved cells, whereas HPi2, an endocrine pan-islet cell marker, was highly expressed in both fresh and cryopreserved epithelial cells, indicating the purity of the islet preparations used and the retention of characteristic surface markers after freeze-thaw. Notably, the fraction of EpCAM-expressing cells significantly reduced immediately after thawing (to 10.9 ± 4.3%, compared to 83.4 ± 6.9% in fresh cells, p = 0.02) (Fig 3B right panel).

As seen in Fig 3B left panel, although the best cryopreservation procedure did not affect viability when cells were assayed immediately after thaw, the percent of live cells reduced to < 50% after overnight culture consistent with the decrease in cell viability as assessed using SYTO 13/GelRed after overnight incubation (Fig 2B). Previously, it was shown that even though there was regional damage to the extracellular matrix structure of the islets after 24 h culture, cytoplasmic, mitochondrial, nuclear and plasma membrane integrity was retained, indicating that the cells were in the reversible stage of cell injury [43]. Thus, it is a reasonable observation in our study that although the EpCAM (CD326) expression decreased immediately after thaw, the expression was recovered after overnight incubation (Fig 3B, right panel). Similar recovery in surface marker expression (CD120b) has been observed in other cells such as regulatory T cells [45]. Taken together, our cryopreservation procedure allows recovery of viable cells immediately post-thaw, while the cell surface markers may change transiently. Although viability is an important measure of cryopreservation efficiency [46], functionality is an equally important consideration after cryopreservation. Here, we examined electrophysiological properties and transcriptome profiles of dispersed islet cells before and after cryopreservation.

### Effect of cryopreservation on electrophysiology of islet cells

**Fig 4:**
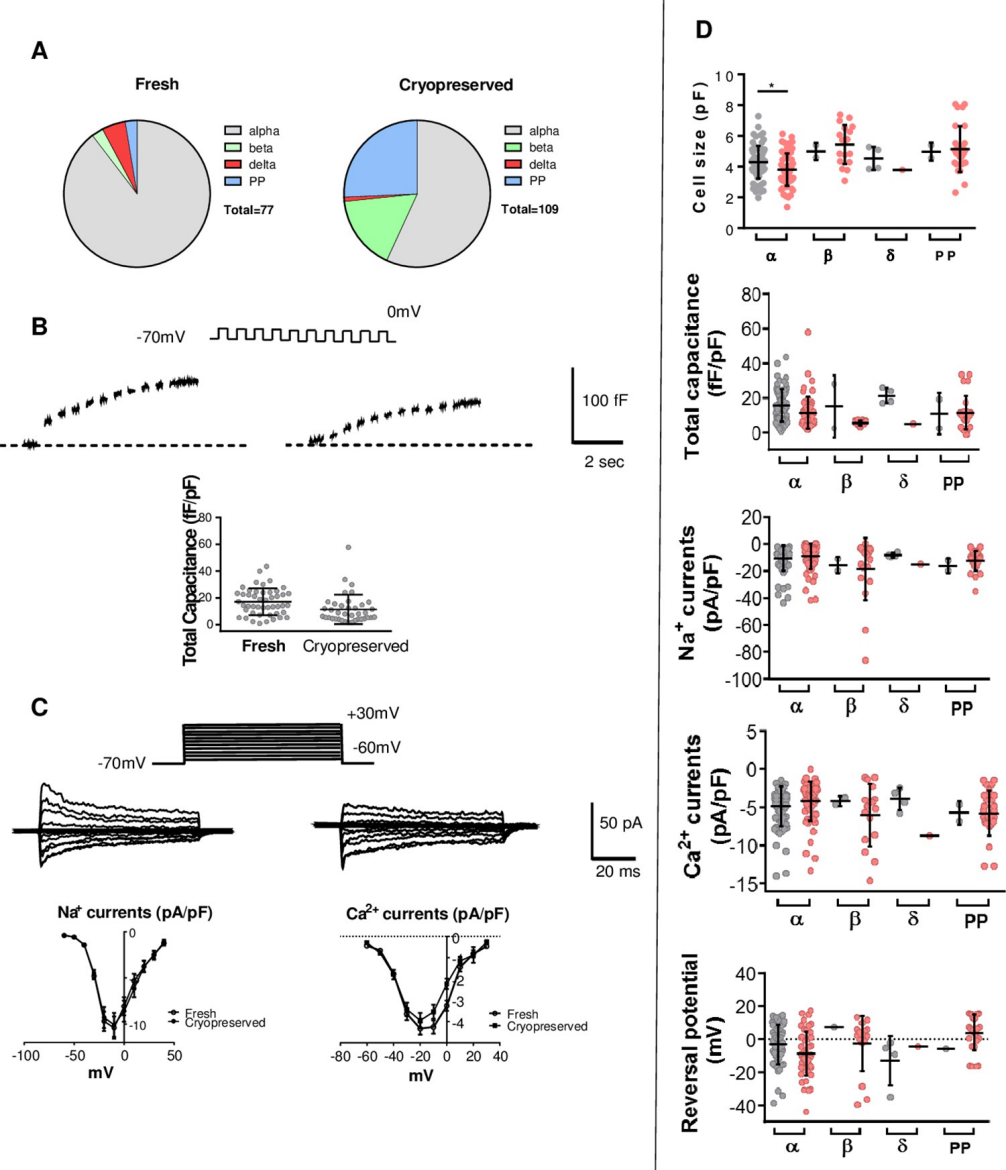
Representative traces from fresh and cryopreserved islet cells showing (A) total numbers of cell types patched; (B) total exocytosis in α cells; (C) top, traces of activation of voltage-gated Na^+^ and Ca^2+^ channel currents in α cells; bottom, the current-voltage relationship of voltage-gated Na^+^ and Ca^2+^ channels; (D) Summary of major electrophysiological data comparing fresh (grey) and cryopreserved (red) dispersed islet α cells, β cells, δ cells, and PP cells from three pancreatic islet donors. Data show cell size, total exocytosis, Na^+^ and Ca^2+^ currents, and reversal potential (S2 Table).

A critical component of islet cell function is the electrical activity mediated by the ion channels, leading to Ca^2+^ influx and the exocytosis of hormone-containing granules. We assessed the function of human single pancreatic islets cells to examine the effects following cryopreservation from 3 days to approximately 2 months, in order to provide a source of high quality dispersed islet cells, and demonstrated the potential for this approach. Fig 4A shows representative data from dispersed islet cells from three donors (R292, R297, and R305 in Table 1). Cryopreservation showed no impact on cell size of most cell types (Fig 4A). Moreover, while the data demonstrate substantial variation in exocytosis and channel activities in different pancreatic islet cell types, electrophysiological parameters of individual cell types were not affected by cryopreservation. These include peak Na^+^ currents (activated at –10 mV), time constant of Na^+^ channel inactivation, peak Ca^2+^ currents (activated at –10 mV, mostly high-voltage activated Ca^2+^ channels), peak Ca^2+^ channel conductance, reversal potential, and hyperpolarization-activated non-selective cation currents (Fig 4D). The data in general, suggested that human single pancreatic islet cells retain many physiologically relevant properties. Critical exocytotic machinery components, e.g., ion channel activities and exocytotic response at 5 mM glucose, are still persistent and sustained. However, some cell functions are indeed reduced in different cell types, e.g., cumulative capacitance and first depolarization-activated capacitance decreased in cryopreserved α cells compared to their fresh counterparts; late depolarization-activated capacitance decreased in cryopreserved α cells, but not in β cells; Ca^2+^ charge entry decreased in cryopreserved α and β cells. Although we observed some minor reduction of granule exocytosis capacity after cryopreservation, the key electrophysiological properties, such as voltage-gated Na^+^, Ca^2+^ channel currents and reversal potential were unaffected.

### Effect of cryopreservation on transcriptome of islet cells

**Fig 5:**
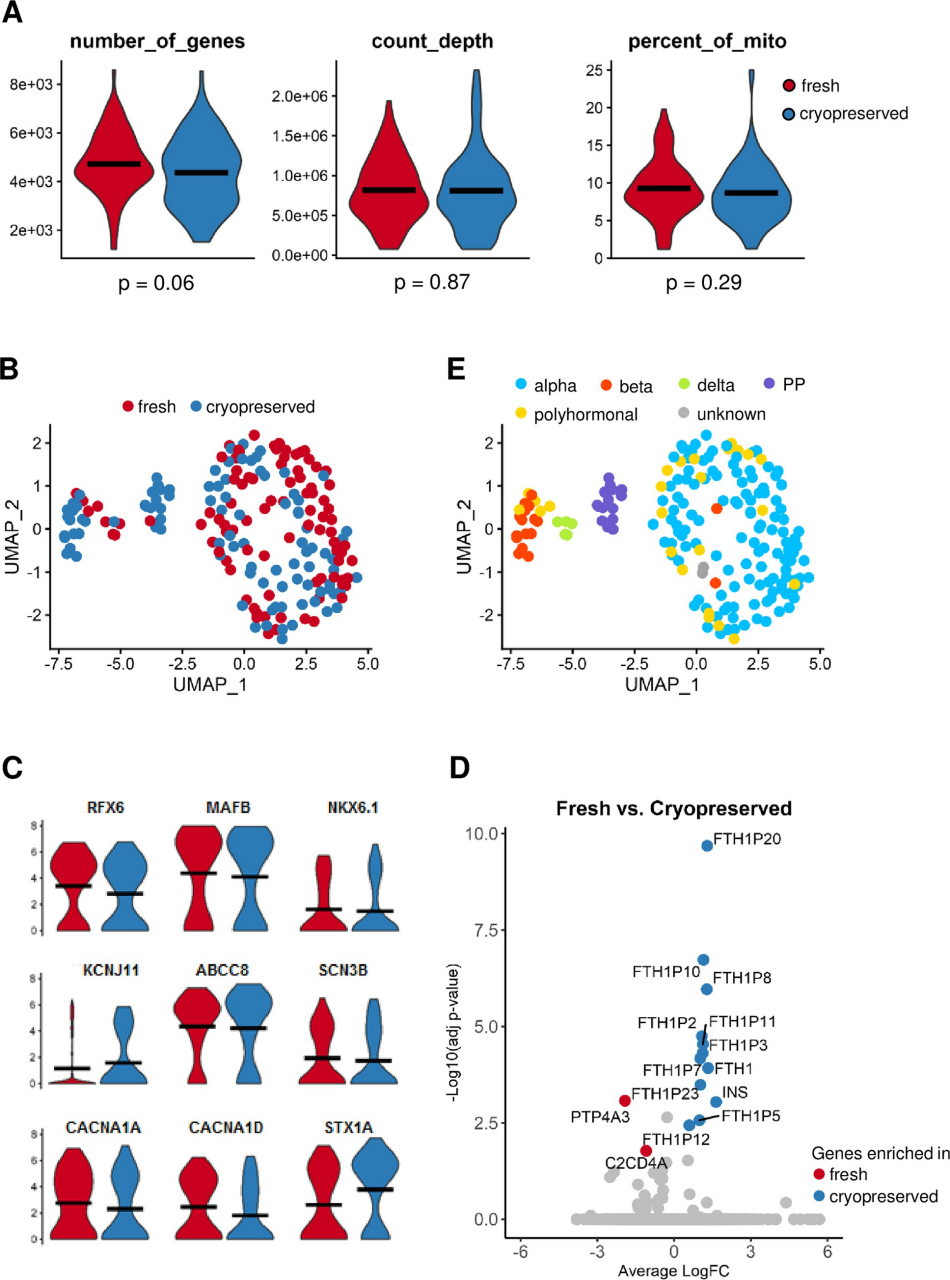
Transcriptome comparison between fresh and cryopreserved cells. In total, 208 patch-clamped single cells were collected to make RNASeq libraries and sequenced, out of which 189 cells passed quality control threshold (1000 < number of genes detected per cell < 10000, count depth > 50000). These include 91 fresh and 98 cryopreserved islets cells. **A) Overall single-cell transcriptome qualities of fresh and cryopreserved cells.** Violin plots depict the distribution of three metrics for single-cell transcriptome assessment, i.e., number of genes detected (left), sequence count of transcripts (middle), and percentage of mitochondrial transcripts of total transcripts (right) in single cells. In each violin plot, fresh cells are shown in red and cryopreserved cells shown in blue. Black bars represent the mean of each assessment, and p-values from Student’s t-tests are listed below the graphs. **(B) Unsupervised clustering analysis of sequenced cells.** Uniform Manifold Approximation and Projection (UMAP) is used to reduce dimensionality and visualize the single-cell transcriptomic data. Each dot represents one single-cell RNASeq library with fresh islet cells in red and cryopreserved islet cells in blue. Fresh or cryopreserved cells are distributed in all clusters in a non-discrete pattern, indicating that cryopreservation-associated transcriptome changes are not the driving force of clustering. **(C) Levels of representative transcripts encoding transcription factors, ion-channels, and secretion machinery in fresh and cryopreserved cells.** Quantifications of genes previously linked to islet cell function are depicted by violin plots. In each violin plot, fresh cells are shown in red and cryopreserved cells shown in blue, with black bars denoting means of expression and gene names listed above the plots. **(D) Differentially expressed genes in fresh and cryopreserved islet cells.** Model-based Analysis of Single-cell Transcriptomic (MAST) [35] is used to identify differentially expressed genes between fresh and cryopreserved islet cells. A volcano plot is used to illustrate the gene distributions, with each dot representing a gene. Genes with significant difference (adjusted p-value < 0.05 and average fold-change > 1.5) are highlighted in red (enriched in fresh cells) and blue (enriched in cryopreserved cells). **(E) Cell type annotation based on canonical markers.** Cells (dots) were colored based on annotated cell types with legend shown on top. All major islet cell types, except ghrelin-producing epsilon cells, were detected in both fresh and cryopreserved cells. Alpha cells account for the majority, with polyhormonal cells with mixed gene signature present.

Patch-seq has enabled investigation of gene profile and functionality of the same single islet cell [30]. Fig 5A shows that overall transcriptome qualities (i.e., number of uniquely mapped reads, number of detected genes, etc.) are comparable between fresh and cryopreserved cells, indicating that the optimized cryopreservation method maintained the quality and stability of transcriptome in dispersed human islet cells (S3 Table). Unsupervised cell clustering analysis revealed fresh and cryopreserved cells cluster in a non-discrete pattern (Fig 5B). Moreover, close examination of the genes that are previously linked to alteration in cell identity or function, such as signature transcription factors (e.g. *PDX1*, *MAFA*, *MAFB*, *NKX6*.*1*, *RFX6*), ion channels (e.g. *KCNJ8*, *ABCC8*, *SCN3A*, *CACNA1A*, *CACNA1D*), and key components of exocytosis machinery (e.g. *STX1A*), revealed no significant differences (Fig 5C). However, differential expression analysis revealed a handful of genes altered between fresh and cryopreserved islet cells (S4 Table). Strikingly, eleven members of the FTH1 gene family encoding the heavy subunits of ferritin were upregulated in cryopreserved cells (Fig 5D), possibly a cell response to anoxia during cryopreservation [47]. Importantly, we confirmed that all major islet cell-types, α-, β-, δ- and PP-cells are represented in this survey (Fig 5E) using a combination of canonical marker genes encoding cell specific hormones and transcription factors (Table 2).

**Table 2:** Canonical islet cell markers used to annotate cell types.

Cell Type	Canonical Markers
alpha	*GCG*, *ARX*, *MAFB*, *IRX2*
beta	*INS*, *PDX1*, *MAFA*, *NKX6*.*1*
delta	*SST*, *HHEX*, *PDX1*
PP	*PPY*, *ETV1*, *IRX2-neg*, *MAFB-neg*
epsilon	*GHRL*

## Discussion

Many attempts have been made to cryopreserve whole intact islets as pancreatic islet transplantation emerged as a promising treatment for Type 1 diabetes [48–50]. Cryogenic banking of whole islets for several years has been reported to maintain limited viability and function [51,52], and improvements in post-thaw cell recovery, function, and transplantation outcome continue to be sought. We have previously employed an iterative optimization approach to achieve significant improvements in the cryopreservation of various tissues and cell types [15–18,53,54]. To the best of our knowledge, this is the first systematic study of the cryopreservation of dissociated islet cells. This work investigates cell-type specific outcomes of the best cryopreservation procedure. We did not consider optimization of the cryopreservation for individual cell types by sorting cells prior to cryopreservation because sorting the cells before cryopreservation could induce physiological changes as demonstrated in astrocytes [55].

Reducing the concentration of cryoprotective agents in the cryopreservation solution has been a strategy employed to minimize their cytotoxic effects and improve recovery [56]. The use of 5% DMSO, 6% HES and 4% human albumin resulted in 71% recovery of human islets with their morphological structure and insulin secretion retained after storage in liquid nitrogen for 2 weeks to 3 months [39]. Another approach involved vitrification, such as that employed for rat pancreatic islets (using 15% DMSO, 15% EG, and 0.5M sucrose) which showed < 60% immediate post-thaw survival [57]. Recently, rat pancreatic islets were preserved at −3°C ± 0.1°C in a rigid constant volume chamber in the absence of cryoprotective agents, and were shown to be 75% viable after 72 h; however, viability gradually declined with time of storage [58]. The methods currently employed for whole intact islet cryopreservation report a loss of 20–40% of recovered viable islets [59] and transplantation of cryopreserved islets have been met with only moderate success [60]. Thus, the cryopreservation of dissociated islet cells may prove to be more successful than cryopreservation of intact islets due to the lower level of complexity.

The main objective of this study is to demonstrate that dispersed islet cells remain viable, and retain their molecular and phenotypic characteristics and function after cryopreservation. In this work, we optimized the cryopreservation of single islet cells in suspension by applying a graded freezing procedure that allowed the identification and mitigation of slow-cooling and rapid-cooling injury. For evaluating the different cryopreservation conditions, we used membrane integrity as the sole post-thaw outcome. Because the plasma membrane is considered to be the primary site of cryoinjury and cryoinjury is typically catastrophic to the cells, membrane integrity is routinely used in cryopreservation optimization and can be considered to give an upper limit in the assessment of cell viability. Therefore, we deemed it sufficient to use membrane integrity during the optimization steps. The procedure that gave the highest membrane integrity post-thaw was used to cryopreserve dispersed islet cells. Then, thawed cells were compared to their unfrozen counterparts in functional assessments including electrical activity by patch clamp electrophysiology, molecular and phenotypic markers by flow cytometry, and gene expression by patch-seq. By using a combination of a permeating cryoprotectant (DMSO), which mainly minimized slow-cooling injury, and a non-permeating cryoprotectant (HES), which mainly minimized rapid-cooling injury [38], we were able to efficiently test the effects of cryoprotectant type and concentrations. The final procedure for cryopreserving dispersed islet cells that we developed involved equilibration with medium containing 10% DMSO and 6% HES for 15 min at 0°C, ice nucleation at –5°C and release of the latent heat of fusion at –5°C for 3 min, cooling at 1°C/min to –40°C, then plunging into liquid nitrogen. The cells were rapidly thawed to prevent small ice crystals, which could have formed during cooling, from growing and subsequently damaging the cells. Membrane integrity was highest when the cryoprotectants were removed by serial dilution.

Recent advances in single-cell genomics have allowed transcriptional profiling of thousands of single cells [61] and enhanced our understanding of the role of cellular heterogeneity in the human islet [22]. Through the process of exocytosis, endocrine cells in the pancreatic islet secrete the hormones insulin, glucagon, somatostatin, pancreatic polypeptide, and ghrelin. Electrical activity is crucial for hormone release and is orchestrated by voltage-gated Na^+^ and Ca^2+^ channels. Exocytosis can be monitored at the single-cell level by using patch-clamp electrophysiology to measure electrical responses mediated by these ion channels. We were able to quantify single cell vesicle exocytosis and ion-channel activity and then recover cellular contents for parallel assessment of gene expression in the same cell, a newly developed protocol known as pancreas patch-seq. We found that the key electrophysiological parameters (exocytosis, Ca^2+^ and Na^+^ currents) from cryopreserved dispersed islet cells had comparable quality metrics to those of fresh cells. Unexpectedly, the main transcriptomic difference detected by single cell sequencing is increased levels of *FTH1* and its 10 pseudogenes in cryopreserved islet cells. *FTH1* encodes the heavy chain subunit of ferritin, while its pseudogenes serve in various forms to regulate *FTH1* expression (reviewed in [62]). Ferritin is traditionally known as the principal intracellular iron storage protein complex that is crucial in cellular iron homeostasis. However, mounting evidence also points to its essential role in protecting DNA from iron-induced oxidative damage in the nucleus [63–65]. *FTH1* is the preferred subunit in the protective nuclear ferritin complex. Hence, our observation of elevated *FTH1* likely reflects an intrinsic mechanism by islet cells to mitigate cryopreservation-induced oxidative stress, a widely-cited causative factor for cryoinjuries [66]. Although beyond the scope of current study, understanding the molecular mechanisms underlying *FHT1* induction during cryopreservation will benefit future development of effective cryoprotective agents.

The utility of cryopreserved individual islet cells extends beyond their use in single-cell genomics and functional studies. Re-aggregation of these cells post-thaw into spheroids enhanced metabolic activity and proved to be an effective approach in achieving normoglycemia in diabetic rats, even better than transplantation of fresh intact islets [14]. The viability was significantly higher (97%) in the cryopreserved islet spheroids compared to cryopreserved native islets (15%), suggesting that pseudo-islet generation after thawing may improve viability. Notably, we observed in both the freshly isolated and dispersed cells and the thawed cells that viability decreased with an additional overnight culture. Although viable post-thawed cells profiled by electrophysiological assessment and scRNA-seq were comparable to freshly isolated cells, re-aggregation following thawing may be an approach worth considering here as the re-aggregation process selects for the healthier cells and excludes the cells that were damaged by cryopreservation [14]. Indeed, in culture the *in vitro* survival and *in vivo* functionality of pseudoislets appear significantly improved over that of the native islets from which they were derived [67,68]. This may recapitulate observations in smaller islets (50–150 μm in diameter) which also appear less sensitive to cryoinjury [69]. By dissociating rat islets into single cells and using a micromold to re-aggregate islets to a maximum diameter of 125 μm, a viability of nearly 100% was achieved, with increased glucose diffusion and insulin secretion relative to native islets [70]. Another study showed that cryopreserved dispersed islet cells can be used to prepare functional islet cell sheets that can potentially be a graft for transplantation into diabetic patients [71]. Multi-layer β cell constructs of controlled thickness on biopolymer films have also been engineered from dispersed islet cells in order to improve mass transport and tissue viability [72]. These studies validate the merit of using single islet cells in suspension, and their cryopreservation would offer a readily available source of cells for research, tissue engineering, or transplantation.

In conclusion, we present a procedure for cryopreserving dispersed islet cells that maintained their membrane integrity, transcriptome, and functional phenotype. Our findings constitute a step forward in providing a readily accessible source of islet cells for single cell genomics and functional studies with the goal of understanding the mechanisms regulating pancreatic islet function, and ultimately designing novel diagnostic and treatment strategies for pancreatic disorders such as diabetes. As isolation of single cells is of paramount importance to these approaches, we expect more studies will explore the use of on-demand cryopreserved islet cells. The assembling of single islet cells into three-dimensional aggregates (pseudoislets or islet spheroids) of specific size or composition has been shown to have beneficial effects on islet survival and function after transplantation [7,8]. Recently, pseudoislets that were fabricated from dispersed rat islet cells that have been cryopreserved were shown to have comparable viability and insulin secretion potential as those prepared from fresh islets [73]. Thus, cryopreserved dispersed islet cells may represent another source of viable cells for bioengineering functional pseudo-islets or islet-sheets for disease modeling studies and potential transplant applications.

## Supporting information

S1 Fig (TIF)

S2 Fig (TIF)

S3 Fig (TIF)

S1 Table Numerical values for data in Figs 1A, 1B, 2A, 2B, and S3. (CSV)

S2 Table Numerical values for data in Fig 4. (XLSX)

S3 Table Metadata of transcriptome analysis (Fig 5A and 5B). (CSV)

S4 Table Differential gene expression (Fig 5D). (CSV)
