# Supplementary figures and images for: Cryopreservation and post-thaw characterization of dissociated human islet cells
Source: PLoS One. 2022 Jan 26;17(1):e0263005. doi: 10.1371/journal.pone.0263005 (PMC8791532; doi:10.1371/journal.pone.0263005)

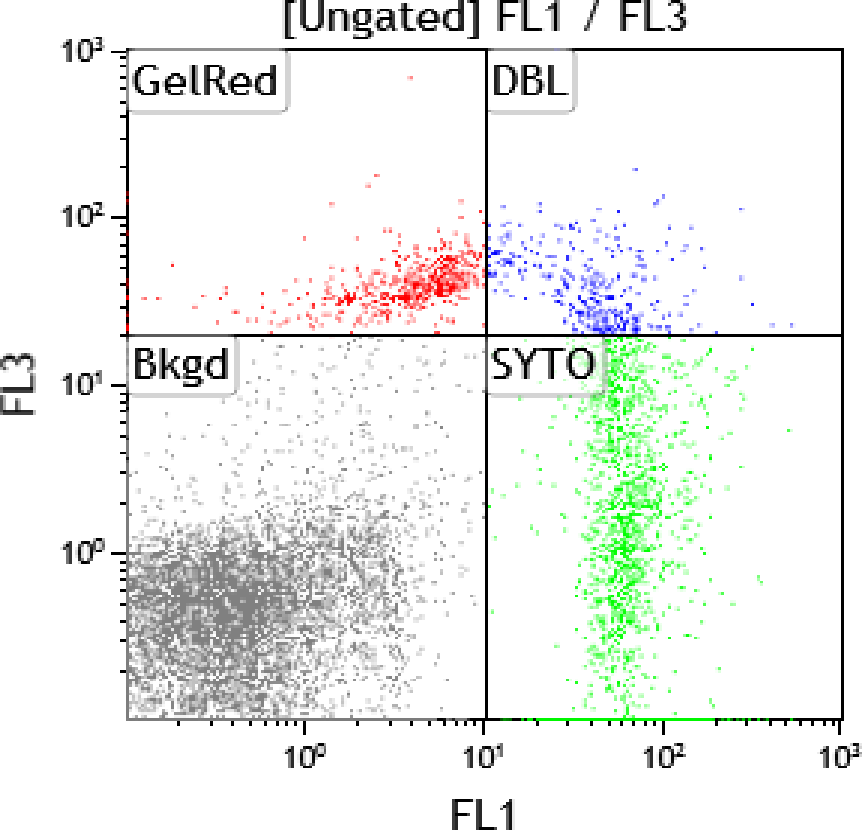

Supplement: S1 Fig — (TIF) [file pone.0263005.s001.tif]

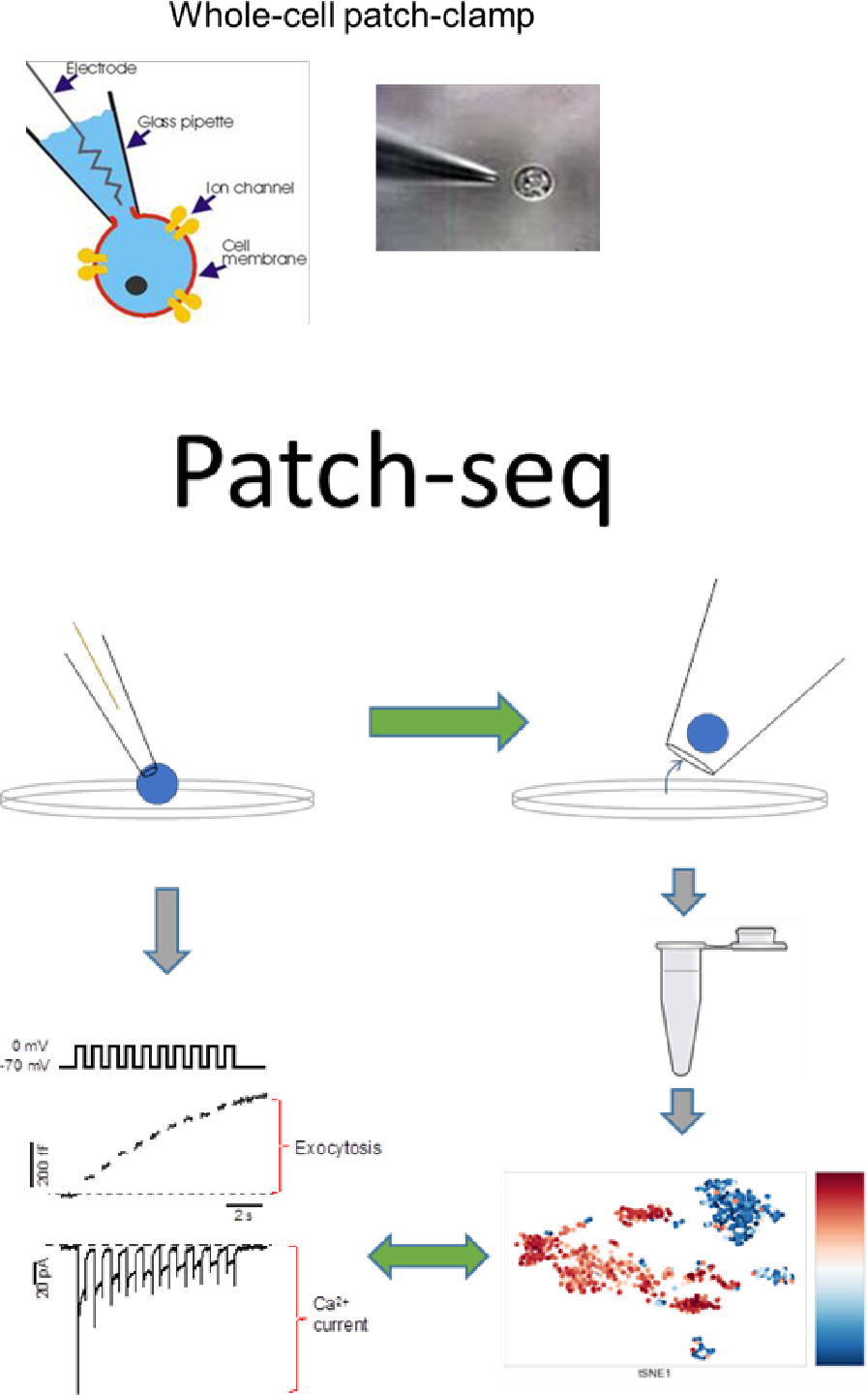

Supplement: S2 Fig — (TIF) [file pone.0263005.s002.tif]

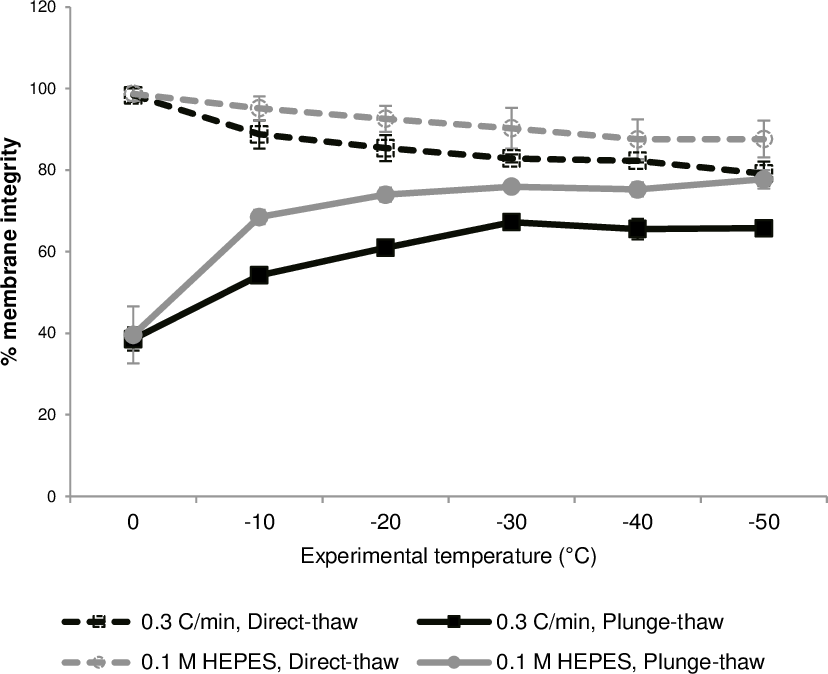

Supplement: S3 Fig — (TIF) [file pone.0263005.s003.tif]
